# Gap between the concerns of healthcare professionals and parents’ perceptions regarding dietary habits for 18-month- and 3-year-old children in Japan

**DOI:** 10.1186/s12889-023-16743-z

**Published:** 2023-09-30

**Authors:** Midori Ishikawa, Yumiko Morinaga, Mayu Haraikawa, Yuka Akiyama, Kemal Sasaki, Saki Horie, Nobuo Yoshiike, Yoshihisa Yamazaki, Tetsuji Yokoyama

**Affiliations:** 1 Department of Health Promotion, National Institute of Public Health, 2-3-6 Minami, Wako, Saitama 351-0197 Japan; 2 Department of Nursing Science, Faculty of Health and Welfare Science, Okayama Prefectural University, 111 Kuboki, Soja-Shi, Okayama 719-1197 Japan; 3 Department of Child Studies, Faculty of Education, Seitoku University, 550 Iwase, Matsudo, Chiba 271-8555 Japan; 4 Department of Health Sciences, School of Medicine, University of Yamanashi, 1110, Shimokato, Chuo, Yamanashi, 409-3898 Japan; 5 Department of Food and Health Sciences, Faculty of Human Life Sciences, Jissen Women’s University, 4-1-1 Osakaue, Hino, Tokyo, 191-8510 Japan; 6 Graduate School of Health Sciences, Aomori University of Health and Welfare, 58-1 Mase, Hamadate, Aomori, 030-8505 Japan; 7 Child Health Center, Aichi Children’s Health and Medical Center, 426-7 Morioka, Obu, Aichi 474-8710 Japan

**Keywords:** Gap, Concerns, Dietary habits, Preschool children, Parents, Healthcare professionals

## Abstract

**Background:**

A gap has been reported between healthcare professionals’ (hereafter “professionals”) recognition of preschool children’s diets and parents’ perception of concern. This study investigated the gap between the concerns reported by professionals and parents’ perceptions regarding health and dietary habits by age (18 months and 3 years) and gender in Japan.

**Methods:**

The study design consisted of a cross-sectional, multilevel survey. The request letters were sent to all households with target children with the cooperation of local governments. After obtaining written informed consent from parents, questionnaires were distributed to them. The survey included 30 items on children's concerns about health and dietary habits. At the health checkup, parents indicated whether they were concerned in response to each item, and responded child’s height and weight and birth height and weight. Next, the professionals provided counseling to the parents at a health checkup. After that, the professionals noted their concerns in response to the same 30 items as those given to parents. The participation rates were 82.9% (18 months) and 82.8% (3 years). Data of 239 persons for 18 months and 223 persons for 3 years old were analyzed. In the statistical analysis, the items that were judged as concerning by professionals but not by parents were identified; likewise, the items that were of concern to parents but not to professionals were identified. Sensitivity, false negative rate, specificity, false positive rate, and Youden index were calculated to analyze the discordance rate for each item.

**Results:**

Many parents in this study were concerned about the issues that professionals did not consider to be concerning. Moreover, the parents worried about more issues for 3-year-olds than for 18-month-olds. The items for which ≥ 10 professionals indicated concerns and with higher discordance between the professionals and parents for both boys and girls were “picky eating” for 18-month-olds and “inconsistent amount of food” for 3-year-olds.

**Conclusions:**

The concerns that professionals have with respect to children's diets and the things that parents worry about show gaps. It might be necessary to provide professional counseling for parents to develop a correct understanding of their children’s dietary habits.

**Supplementary Information:**

The online version contains supplementary material available at 10.1186/s12889-023-16743-z.

## Background

During early childhood, early involvement in the formation of children’s diet and food behaviors affects later development and health [1–5]. To support good growth in children, healthcare professionals (hereafter “professionals”) need to identify concerns about children’s health, nutrition, diet, and food habits during health checkups [6, 7] and to provide necessary nutritional counseling [8–10].

If professionals are to respond appropriately to children’s nutrition challenges, it is necessary to accurately respond to parents’ concerns regarding their children’s health and dietary habits and to identify the matters of concern that are important in child development but tend to be overlooked [9–11].

If parents or guardians misperceive something about their child’s eating or food habits of their child or misunderstand the implications of something, they need to be provided with accurate guidance during childcare/nutrition counseling by professionals. Parents’ knowledge and skills must be modified to positively influence the quality of their child’s diet [6, 12, 13]. In addition, parents’ ability to perceive their children’s health and nutrition correctly brings them a sense of security.

In Japan, the Maternal and Child Health Act requires all municipalities to implement health checkups for preschool children aged 18 months (children over the age of 18 months and under the age of 2 years) and 3 years (children over the age of 3 years and under the age of 4 years) [14]. As part of this process, professionals (including dietitians, public health nurses, and other professionals) can come to better understand parents' concerns regarding their children's health and feeding and provide them with the materials to help them form appropriate food habits. Professionals also provide follow-up consultations to enable parents to feel more secure, if necessary [14, 15]. However, the parents may not be concerned about some matters in their child’s eating or diet that a professional might consider concerning. It is important for effective childcare and nutrition counseling to close the gap between what professionals consider concerning and what parents do.

Some studies on the dietary habits of young children have investigated items that the professionals consider concerning and that make parents worried [15–18]. Previous studies have reported that picky eating stemming from unbalanced meals can be an issue of concern for young children, but that there tends to be a gap in the recognition of it between professionals and parents [19, 20]. However, few reports have clarified the differences between the professionals’ concerns and parental worries by age and gender in preschool children.

There have also been few studies to identify differences between the concerns of professionals and the perceptions of parents with reference to the feeding of preschool children in Japan. In studies of support parents and children, a gap has been seen between parents and professionals, where parents may be worried about things that professionals do not consider a concern, as well as the reverse, where parents are not worried about things that professionals consider a concern.

This study was undertaken to compare the concerns of professionals and those of parents regarding the health and dietary habits of 18-month- and 3-year-old preschool children and identify the gap between the two. From the results of this study, childcare and nutrition counseling materials created by dietitians and public health nurses can be provided to parents.

## Methods

The subjects of the survey for this study were (1) parents of 18-month- and 3-year-old children who agreed to participate in the survey and (2) healthcare professionals (public health nurses, registered dietitians, etc.) who provided childcare and nutrition counseling for those parents at health checkups and who agreed to participate in the survey.

First, local governments that would be able to cooperate with this study in municipalities with different population sizes in various regions of Japan were searched, as the number of children born and their living environments differ by region. The purpose of the study was presented to the municipalities’ governments, and their assistance and cooperation in the study were requested. The purpose and specific details of the surveys were carefully explained to the staff members in charge of health checkups for the 18-month- and 3-year-old children, and their cooperation was requested. Following this, a research cooperation agreement was signed with the municipalities that agreed with this study's objectives and content, allowing a survey of both parents and health professionals. Written consent was obtained from three municipal governments.

Ultimately, the study was carried out with the cooperation of three municipalities, one in the Tohoku region (A Town), one in the Chubu region (B Town), and one in the Chugoku region (C City). A Town is an agricultural/fishing rural area in the north part of Japan, B Town is an industrial urban area in the central part of Japan, and C City is a historically commercial urban area in the west part of Japan. (Because the study asked parents about their concerns with respect to their children and the judgments of professionals, due to careful ethical considerations, the names of specific cities and towns are not provided here.)

The survey period was from March 2019 to January 2020, and it was conducted on days when it was possible to coordinate survey administration with health checkups in municipalities.

### Survey for parents at child health checkups in local government

In Japan, the Ministry of Health, Labor and Welfare (MHLW) has been developing and revising the guidelines for the standards for measurement methods and practical manuals for specialists regarding health checkups for infant based on the Maternal and Child Health Act [14] by MHLW research program grant [21].

From these guidelines, all local governments have developed criteria for the assessment of children at health checkups.

Furthermore, in standard health checkups, before or after the checkup, a multi-professional meeting is convened by health staff to identify health concerns (a “pre-/post-conference”). Although this form of conference is not mandated, this form has been adopted for information sharing among staff to connect health checks and health guidance for children in local governments and to implement practical training for measurement.

At the pre-conference, the staff discusses pre-established concerns concerning each child. At the post-conference, the children who require follow-up evaluations were confirmed. In some cases, continuous support (follow-up) [15, 22, 23] and the provision of nutritional guidance in conjunction with community collaborations may be required. This sharing allows the best approaches for supporting children and parents to be decided, responses to their needs to be evaluated, and the outcomes of those activities to be assessed [15, 22]. In this study, due to this system in local governments, inter-individual bias in judgments between professionals was considered to be minimal.

#### Target households

**Fig. 1:**
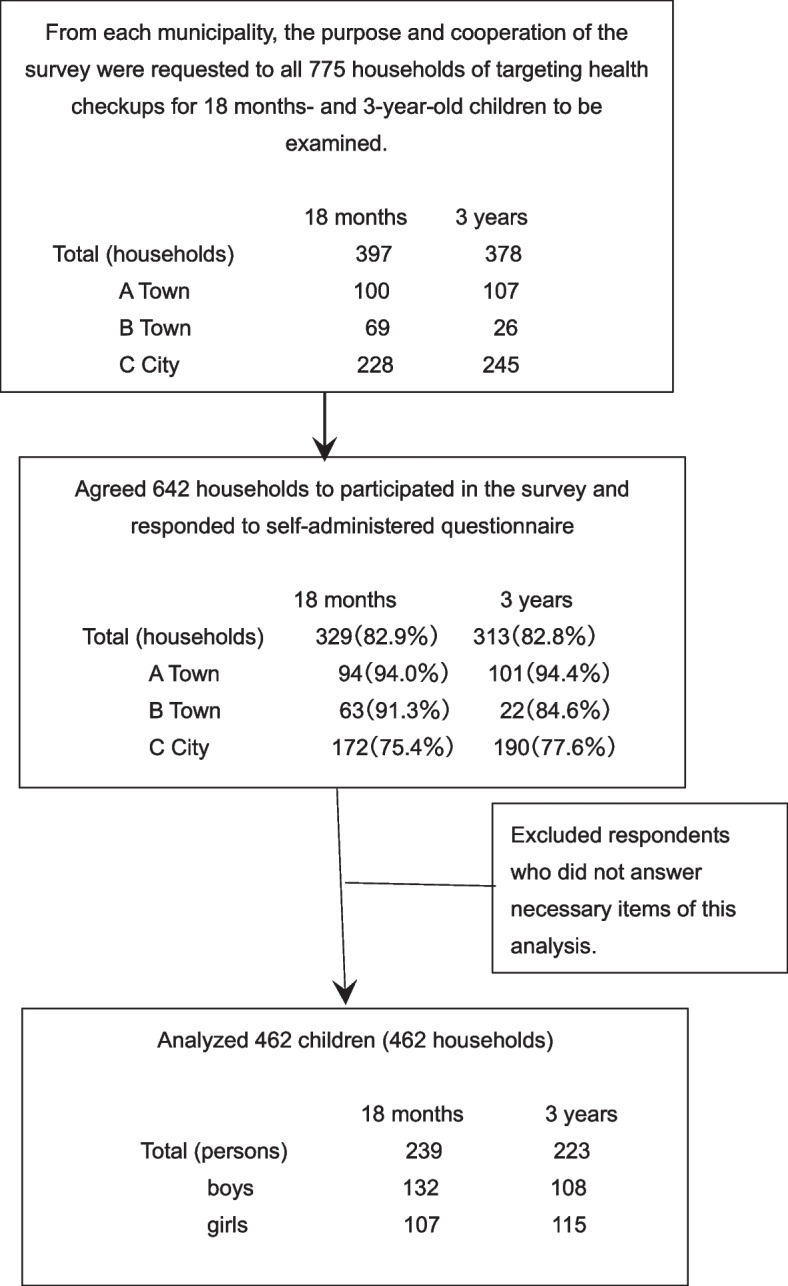
Study population and procedure diagram of this study

The written consent and a completed survey were obtained from 329 households (94 households in A Town, 63 in B Town, and 172 in C City) with 18-month-old children, out of 397 households (100, 69, and 228, respectively), for a cooperation rate of 82.9%. The written consent and a completed survey were obtained from 313 households (101 households in A Town, 22 in B Town, and 190 in C City) with 3-year-old children, out of 378 (107, 26, and 245, respectively). The cooperation rate was 82.8% (Fig. 1). The parents completed the questionnaire describing concerns about their child’s health and dietary habits before the health checkups.

#### Survey items

The survey items were indicators that have been confirmed to be reliable from the National Nutrition Survey on Preschool children [24] and the health and nutritional status and dietary guidance at health checkups for children in Japan [25], as well as acknowledged reliable indicators for identifying nutrition and dietary issues [26–28]. The survey items also drew on the references for infant and young child nutrition provided by WHO [29, 30].

##### Measurement child concerns

The questionnaire included 30 items of potential concern about a child’s health, dietary and food habits, in the following categories: health awareness and lifestyle (10 items), diet content and atmosphere (8 items), interest and motivation in food (8 items), and food experience and behavior (4 items) [18, 31].

The parents were asked if they were concerned about these items, replying “yes” or “no” to each.

Items on health awareness and lifestyle (10 items) included the concerns on “bedtime/wake-up time,” “lack of control over types and amounts of beverages (including sweet drinks),” “snack intake, frequency and time,” “did not understand what meals their child is eating at nursery school” or were “unable to manage the types and amounts of snacks (including sweets).”

For rating the child’s diet content and atmosphere, eight items were given, including “the type and combination of food and ingredients are unbalanced,” “the type and combination of dishes (staple food, main dish, side dish) are not good,” “the arrangements and color of food is not good,” and “the parent was not good at cooking meals.”

Items for the child's interest in and motivation in food (eight items) included “the amount of food my child eats is always small,” “my child is not hungry at mealtimes,” “his or her eating habits are not constant,” “my child eats sluggishly (it takes a long time to eat),” “my child plays with his or her food (lazy eating),” “picky eating (unbalanced diet),” and “irregular mealtimes.”

The items for food experience and behavior (four items) included “not allowing children to experience preparing meals (helping)” and “not allowing them to experience the cultivating and harvesting of ingredients.”

The following information was also requested: the place of residence, relationship with the child, gender of the child, height and weight at birth of the child, order of birth of the child, current height and weight of the child, mother's employment status, child’s daytime caregiving status, and household’s subjective economic conditions and leisure time (Supplementary information).

##### Nutritional status and familial situation of the children

The child’s height, weight, birth height, birth height, and weight and birth order were obtained from the parents.

They provided the value of height and weight professionally measured at the health checkups and the value of birth height and weight and birth order of the child written in the Maternal and Child Health Handbook [21–23]. The nutritional status of children was determined by body weight and height. In addition, the parents stated the location of childcare during the day (nursery school, kindergarten, centers for early childhood education and care, grandparents and other relatives, others, none of the above, and multiple answers allowed), age of parents (mother and father), cohabitants (mother, father, grandmother, grandfather, younger brother or sister, older brother or sister), employment of the child’s mother (yes or no), subjective economic lifestyle (affluent, somewhat, neutral, not well off, unable to afford the cost of living, do not want to answer) and leisure time in the lifestyle (affluent, somewhat, neither, not so much, unable to afford at all, and do not want to answer).

### Cooperation of the healthcare professionals in the study

Professionals (public health nurses and registered dietitians) who were in charge of childcare and nutrition guidance for 18-month-old and 3-year-old infants at the health checkups responded individually to the study. In all, 36 professionals participated in this study: 9 in A Town (8 public health nurses and 1 registered dietitian), 8 in B Town (7 public health nurses and 1 registered dietitian), and 19 in C City (14 public health nurses and 5 registered dietitians).

After the parents completed the questionnaire describing concerns about their child’s health and dietary habits before the health checkups, the professionals provided counseling to the parents. After this counseling, the professionals noted their concerns in response to the same items as those provided to parents. In other words, the professionals indicated whether they shared the parents’ concerns.

### Statistical analysis

Data including all of the items required for this study were analyzed.

First, for the 30 question items, some children had one or more items marked as concerning by health professionals, and some had none. Therefore, the children were divided into two groups (one group with concerns and one without). The situations of children and their families in both groups were compared, including the child’s height, weight, BMI, degree of obesity [32, 33], birth order, birth height, birth weight, daytime care, parents’ age, cohabitants, current employment of the child’s mother, subjective economic conditions, and leisure time. For each item, the number of children marked as concerning by professionals was registered, with the same measure being made for the parents. The results were categorized by age and gender. The category with the most items that professionals indicated concerning was identified.

To clarify the differences between the professionals' areas of concern and parents' perceptions, sensitivity, false negative rate (FNR: 1 − sensitivity), specificity, and false positive rate (FPR: 1 − specificity) were calculated for each item.

Sensitivity is the proportion of parents who were worried about an item for which professionals were also concerned. FNR is the proportion of parents who were not worried about an item for which professionals were concerned.

Specificity is the proportion of parents who were not worried about an item for which professionals were also not concerned. FPR is the proportion of parents who were worried about an item for which professionals were not concerned.

The items for which more than half the parents were not worried, but professionals were concerned (FNR > 0.5) were identified. Moreover, items for which a high proportion of parents were worried about, but professionals were not concerned (FPR > 0.2) were identified.

Finally, the Youden index (sensitivity + specificity − 1) was calculated as a summary index of the differences in concern between professionals and parents. The closer the Youden index is to 1, the more the two groups were in agreement. The items with a high degree of disagreement between the professionals and parents (Youden index < 0.5) were identified as reference values.

All statistical analyses were performed using SAS software, version 9.4 (SAS Institute, Inc., Cary, NC, USA). A *p*-value of < 0.05 was considered statistically significant.

## Results

### Children’s nutritional status and family situation in with/without concerns groups by professionals

Table 1 shows the nutritional status and daytime childcare (adjusted for municipalities) in both the groups who had at least one item of concern by professionals (a group with concerns) and who were assessed as having no concerns (a group without concerns) for 18-month-old children. In the group of boys with professional concerns, mean birth height (*p* = 0.033) and birth weight (*p* = 0.034) were lower and the proportion of those entrusted to grandparents and relatives (*p* = 0.010) was higher than those in the group without concerns.

**Table 1:** Comparison of children’s nutritional status in groups with and without concerns diagnosed by healthcare professionals (18 months old)

			Boys (*n* = 132)					Girls (*n* = 107)				
	Group by diagnose of health professionals		With concerns (*n* = 94)		Without concerns (*n* = 38)			With concerns (*n* = 65)		Without concerns (*n* = 42)		
			mean	SD	mean	SD	*p*^†^	mean	SD	mean	SD	*p*†
Nutritional status	Height (SD score)^a^		− 0.33	1.30	− 0.32	1.06	0.782	− 0.52	1.07	− 0.31	0.94	0.158
	Weight (SD score)^a^		0.03	0.93	− 0.17	1.32	0.426	0.01	1.16	0.09	0.99	0.223
	BMI (SD score)^a^		0.35	1.12	0.52	1.61	0.504	0.49	1.01	0.38	1.05	0.780
	Obese degree (%)^a^		2.08	8.15	4.12	16.93	0.374	2.47	8.46	1.87	8.43	0.604
	Birth height (cm)^a^		49.5	2.1	50.3	1.9	0.033	49.1	2.1	49.8	2.2	0.117
	Birth weight (g)^a^		3065.4	365.9	3203.2	344.9	0.034	3018.4	402.7	3084.7	408.2	0.288
			number	%	number	%	*p*^‡^	number	%	number	%	*p*^‡^
	Birth order	1	44	46.8	14	36.8	0.305	29	44.6	21	50.0	0.350
		2	32	34.0	16	42.1		22	33.9	15	35.7	
		3	16	17.0	6	13.2		12	18.5	5	11.9	
		4	2	2.1	2	5.3		0	0.0	0	0.0	
		5	0	0.0	1	2.6		1	1.5	1	2.4	
		6	0	0.0	0	0.0		1	1.5	0	0.0	
			number	%	number	%	*p*^‡^	number	%	number	%	*p*^‡^
Childcare during the day. (Multiple answers allowed)	Nursery school		48	51.1	21	55.3	0.814	37	56.9	22	52.4	0.984
	Kindergarten		0	0.0	0	0.0	-	1	1.5	0	0.0	0.356
	Centers for early childhood education and care		11	11.7	5	13.2	0.323	11	16.9	3	7.1	0.687
	Grandparents and relatives		11	11.7	0	0.0	0.010	3	4.6	4	9.5	0.440
	Others		1	1.1	2	5.3	0.123	3	4.6	0	0.0	0.160
	None of the above		33	35.1	11	29.0	0.431	18	27.7	16	38.1	0.876
Number of the items with concerns by professionals			mean	SD				mean	SD			
			1.5	1.7	-	-		2.0	2.4	-	-	

*p*^†^adjusted for municipalities by analysis of covariance (ANCOVA)

*p*^‡^adjusted for municipalities by Cochran-Mantel–Haenszel test

^a^Continuous variable

Table 2 shows the nutritional status and daytime child care (adjusted for municipalities) for 3-year-old boys and girls in groups with and without professionals’ concerns. In both boys (*p* = 0.019) and girls (*p* = 0.010), the proportions of the number of children with professional concerns were higher in the first-born children than in the second- and subsequent- born children.

**Table 2:** Comparison of children’s nutritional status in groups with and without concerns diagnosed by healthcare professionals (3 years old)

			Boys (*n* = 108)					Girls (*n* = 115)				
	Group by diagnose of health professionals		With concerns (*n* = 70)		Without concerns (*n* = 38)			With concerns (*n* = 76)		Without concerns (*n* = 39)		
			mean	SD	mean	SD	*p*^†^	mean	SD	mean	SD	*p*^†^
Nutritional status	Height (SD score)^a^		− 0.20	0.99	− 0.17	0.95	0.682	− 0.16	0.93	− 0.27	0.92	0.802
	Weight (SD score)^a^		− 0.15	0.88	− 0.10	0.96	0.492	0.13	1.00	0.01	0.92	0.553
	BMI (SD score)^a^		0.05	0.93	0.10	0.97	0.557	0.33	0.97	0.24	1.09	0.482
	Obese degree (%)^a^		− 0.21	7.21	0.28	7.88	0.482	3.07	9.73	2.23	9.51	0.555
	Birth height (cm)^a^		49.4	2.0	50.0	3.4	0.559	48.8	3.3	49.3	1.7	0.538
	Birth weight (g)^a^		2997.5	482.1	3067.7	414.6	0.343	2994.2	421.5	3051.2	365.2	0.271
			number	%	number	%	*p*^‡^	number	%	number	%	*p*^‡^
	Birth order	1	36	51.4	14	36.8	0.019	49	56.6	12	30.8	0.010
		2	21	30.0	14	36.8		23	30.3	16	41.0	
		3	8	11.4	7	18.4		7	9.2	11	28.2	
		4	5	7.1	3	7.9		3	4.0	0	0.0	
		5	0	0.0	0	0.0		0	0.0	0	0.0	
		6-	0	0.0	0	0.0		0	0.0	0	0.0	
			number	%	number	%	*p*^‡^	number	%	number	%	*p*^‡^
Childcare for the child during the daytime. (Multiple answers allowed)	Nursery school		46	65.7	16	55.2	0.760	54	71.1	20	51.3	0.238
	Kindergarten		10	14.3	2	6.9	0.362	4	5.3	2	5.1	0.702
	Centers for early childhood education and care		7	10.0	1	3.5	0.589	10	13.2	2	5.1	0.624
	Grandparents and relatives		3	4.3	4	13.8	0.548	5	6.6	1	2.6	0.172
	Others		2	2.9	0	0.0	0.558	1	1.3	2	5.1	0.582
	None of the above		6	8.6	9	31.0	0.059	7	9.2	12	30.8	0.094
Number of the items with concerns by professionals			mean	SD				mean	SD			
			2.2	2.6	-	-		2.2	2.5	-	-	

*p*^†^adjusted for municipalities by analysis of covariance (ANCOVA)

*p*^‡^adjusted for municipalities by Cochran-Mantel–Haenszel test

^a^Continuous variable

Tables 3 and 4 present the family situation of children (adjusted for municipalities) in groups with and without professional concerns. There were no significant differences in the situations between the groups of 18-month-old boys and girls. In the group of 3-year-old girls with professional concerns (*p* = 0.013), the proportion of those who had an older brother/sister was higher than those in the group without concerns.

**Table 3:** Comparison of children’s familial situation in groups with and without concerns diagnosed by healthcare professionals (18 months old)

		Boys (*n* = 132)					Girls (*n* = 107)				
	Group by diagnose of health professionals	With concerns (*n* = 94)		Without concerns (*n* = 38)			With concerns (*n* = 65)		Without concerns (*n* = 42)		
		mean	SD	mean	SD	*p*^†^	mean	SD	mean	SD	*p*^†^
Age of parents^a^	Age of mother (years old)	32.9	4.8	32.2	4.8	0.595	32.6	4.6	33.1	3.9	0.832
	Age of father (years old)	34.5	6.0	33.5	4.8	0.526	34.7	5.5	35.7	4.5	0.352
		number	%	number	%	*p*^‡^	number	%	number	%	*p*‡
Cohabitants	Mother	87	92.6	35	92.1	0.995	61	93.9	39	92.9	0.854
	Father	91	96.8	37	97.4	0.989	64	98.5	42	100.0	0.597
	Grandmother	26	27.7	9	23.7	0.969	18	27.7	6	14.3	0.496
	Grandfather	23	24.5	7	18.4	0.714	16	24.6	6	14.3	0.965
	Younger brother or sister	7	7.5	4	10.5	0.507	2	3.1	0	0.0	0.447
	Older brother or sister	48	51.1	23	60.5	0.171	36	55.4	21	50.0	0.574
		number	%	number	%	p^‡^	number	%	number	%	p^‡^
Is child’s mother currently employed?	Yes	59	62.8	26	68.4	0.573	46	70.8	25	59.5	0.541
	No	35	37.2	12	31.6		19	29.2	17	40.5	
		number	%	number	%	*p*^‡^	number	%	number	%	*p*^‡^
Subjective economic lifestyle	Affluent	14	14.9	5	13.2	0.873	7	10.8	8	19.1	0.175
	Somewhat	32	34.0	14	36.8		21	32.3	14	33.3	
	Neither	24	25.5	8	21.1		17	26.2	11	26.2	
	Not so much	14	14.9	9	23.7		14	21.5	7	16.7	
	Unable to afford at all	8	8.5	2	5.3		4	6.2	1	2.4	
	Do not want to answer	2	2.1	0	0.0		2	3.1	1	2.4	
Leisure time in lifestyle	Affluent	13	13.8	5	13.2	0.355	7	10.8	4	9.5	0.439
	Somewhat	33	35.1	9	23.7		13	20.0	9	21.4	
	Neither	18	19.2	6	15.8		14	21.5	10	23.8	
	Not so much	25	26.6	14	36.8		24	36.9	15	35.7	
	Unable to afford at all	4	4.3	4	10.5		6	9.2	4	9.5	
	Do not want to answer	1	1.1	0	0.0		1	1.5	0	0.0	

*p*^†^adjusted for municipalities by analysis of covariance (ANCOVA)

*p*^‡^adjusted for municipalities by Cochran-Mantel–Haenszel test

^a^Continuous variable

**Table 4:** Comparison of children’s familial situation in groups with and without concerns diagnosed by healthcare professionals (3 years old)

		Boys (*n* = 108)					Girls (*n* = 115)				
	Group by diagnose of health professionals	With concerns (*n* = 70)		Without concerns (*n* = 38)			With concerns (*n* = 76)		Without concerns (*n* = 39)		
		mean	SD	mean	SD	*p*^†^	mean	SD	mean	SD	*p*^†^
Age of parents^a^	Age of mother (years old)	34.0	5.1	34.6	5.2	0.631	32.9	4.5	35.1	4.4	0.085
	Age of father (years old)	35.5	0.8	36.2	1.1	0.534	35.0	6.3	36.3	5.7	0.340
		number	%	number	%	*p*^‡^	number	%	number	%	*p*^‡^
Cohabitants	Mother	66	94.3	35	92.1	0.838	73	96.1	35	89.7	0.241
	Father	68	97.1	37	97.4	0.911	74	97.4	37	94.9	0.697
	Grandmother	15	21.4	2	5.3	0.378	20	26.3	5	12.8	0.602
	Grandfather	9	12.9	2	5.3	0.823	14	18.4	2	5.1	0.227
	Younger brother or sister	28	40.0	12	31.6	0.280	31	40.8	11	28.2	0.434
	Older brother or sister	35	50.0	23	60.5	0.052	32	42.1	26	66.7	0.013
		number	%	number	%	*p*^‡^	number	%	number	%	*p*^‡^
Is child’s mother currently employed?	Yes	50	71.4	26	68.4	0.867	56	73.7	28	71.8	0.534
	No	20	28.6	12	31.6		20	26.3	11	28.2	
		number	%	number	%	*p*^‡^	number	%	number	%	*p*^‡^
Subjective economic lifestyle	Affluent	12	18.6	9	7.9	0.282	8	10.5	7	18.0	0.548
	Somewhat	19	27.1	10	26.3		20	26.3	6	15.4	
	Neither	18	25.7	14	36.8		27	35.5	15	38.5	
	Not so much	15	21.4	10	26.3		16	21.1	9	23.1	
	Unable to afford at all	4	5.7	1	2.6		3	4.0	1	2.6	
	Do not want to answer	1	1.4	0	0.0		2	2.6	1	2.6	
Leisure time in lifestyle	Affluent	7	10.0	2	5.3	0.833	6	7.9	6	15.4	0.642
	Somewhat	21	30.0	10	26.3		19	25.0	11	28.2	
	Neither	9	12.9	9	23.7		19	25.0	7	18.0	
	Not so much	28	40.0	16	42.1		27	35.5	10	25.6	
	Unable to afford at all	5	7.1	1	2.6		5	6.6	5	12.8	
	Do not want to answer	0	0.0	0	0.0		0	0.0	0	0.0	

*p*^†^adjusted for municipalities by analysis of covariance (ANCOVA)

*p*^‡^adjusted for municipalities by Cochran-Mantel–Haenszel test

^a^Continuous variable

### The number of children with or without professionals’ concerns about each item and whether the parents were worried about the item

**Fig. 2:**
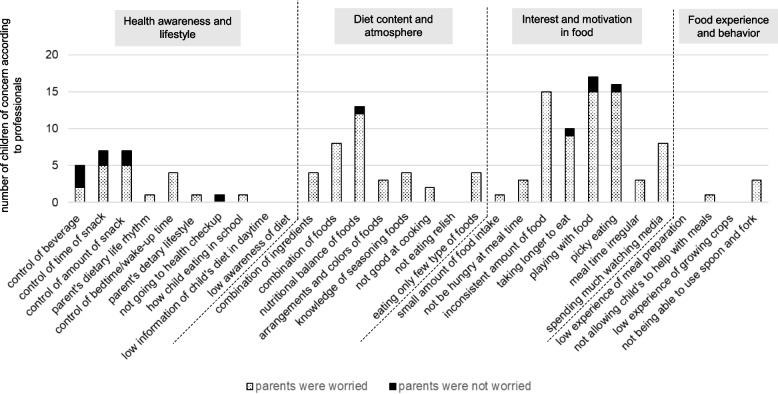
The number of children of concern according to professionals and the number of children whose parents were worried about each item in 18-month-old boys

**Fig. 3:**
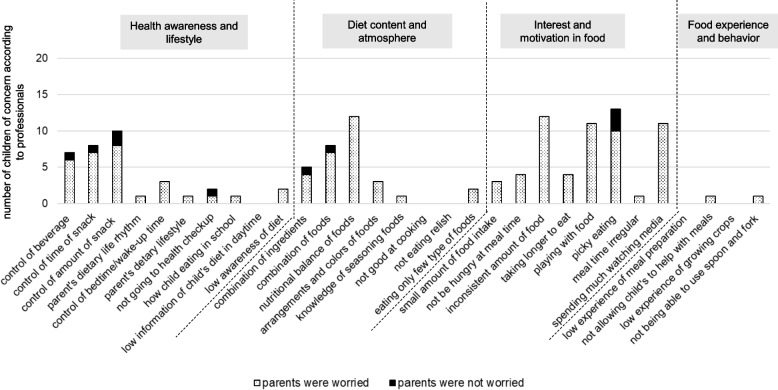
The number of children of concern according to professionals and the number of children whose parents were worried about each item in 18-month-old girls

Figures 2 and 3 show the number of children for whom professionals were concerned and the number of children whose parents were concerned about each item in 18-month-old boys and girls. For the boys (Fig. 2), many items related to “Interest and motivation in food” were noted as concerns by professionals. Among these, some parents were not worried about “playing with food” and “picky eating.” Similarly, for girls (Fig. 3), many items related to “Interest and motivation in food” were noted as concerns by professionals, and some parents were not concerned with “picky eating.”

**Fig. 4:**
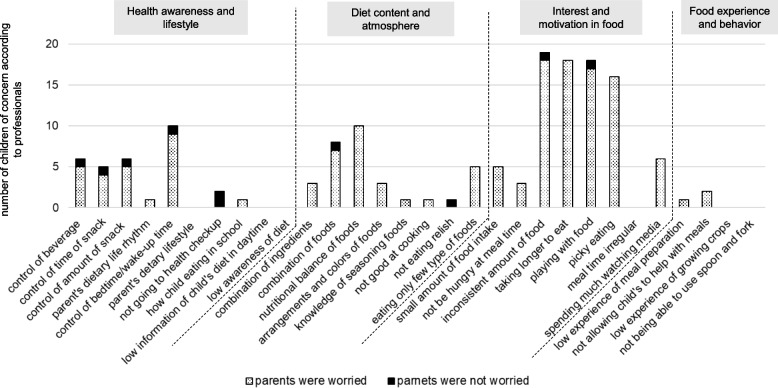
The number of children of concern according to professionals and the number of children whose parents were worried about each item in 3-year-old boys

**Fig. 5:**
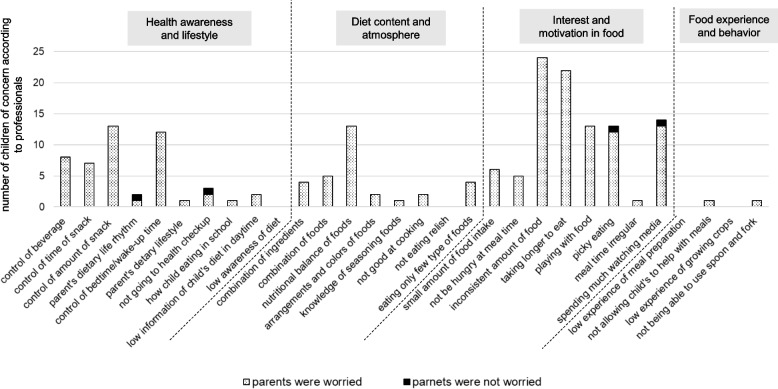
The number of children of concern according to professionals and the number of children whose parents were worried about each item in 3-year-old girls

Figures 4 and 5 present the number of children for whom professionals noted concerns and the number of children whose parents were concerned about each item in 3-year-old boys and girls. For boys (Fig. 4), many items related to “Interest and motivation in food” were noted as concerns by professionals. Some parents were not concerned about the "inconsistent amount of food" and “playing with food.” For girls (Fig. 5), many items related to “Interest and motivation in food’’ were indicated as concerns by professionals, and some parents were not concerned about “picky eating.”

**Fig. 6:**
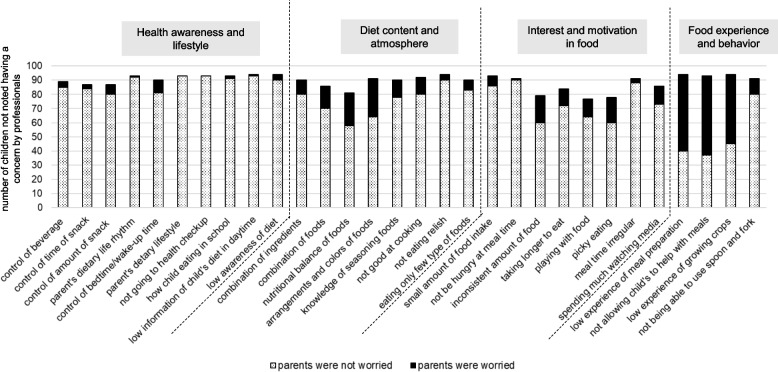
The number of children not noted having a concern by professionals and the number of children whose parents were worried about each item in 18-month-old boys

**Fig. 7:**
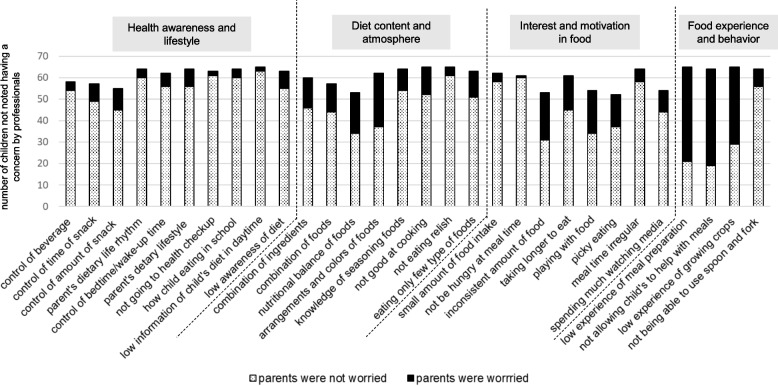
The number of children not noted having a concern by professionals and the number of children whose parents were worried about each item in 18-month-old girls

Figures 6 and 7 present the number of children not noted as having professionals’ concerns and the number of children whose parents were concerned about each item in 18-month-old boys and girls. For boys (Fig. 6), although many items related to “Food experience and behavior” were not considered as concerns by professionals, among them, a large proportion of parents were worried about “low experience of meal preparation,” “not allowing child to help with meals,” and “low experience of growing crops.” For girls (Fig. 7), many items related to “Food experience and behavior” were also recorded by professionals as having no concerns. However, a large proportion of parents were worried about “low experience of meal preparation,” “not allowing child to help with meals,” and “low experience of growing crops.”

**Fig. 8:**
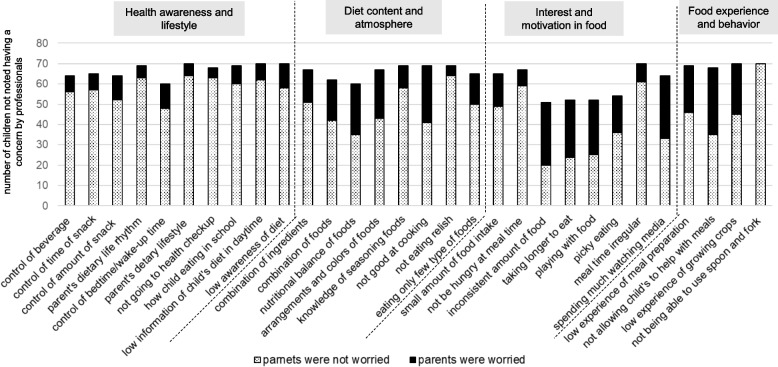
The number of children not noted having a concern by professionals and the number of children whose parents were worried about each item in 3-year-old boys

**Fig. 9:**
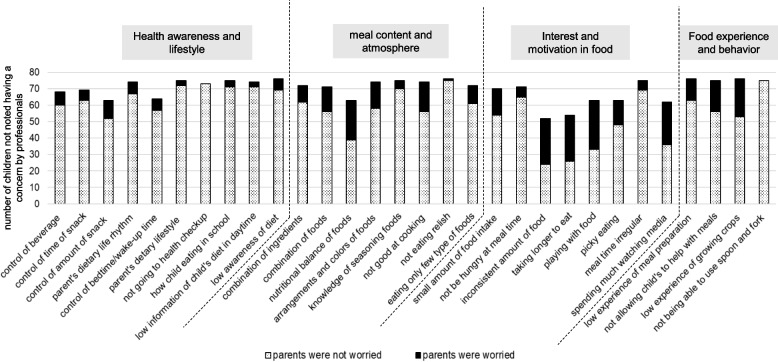
The number of children not noted having a concern by professionals and the number of children whose parents were worried about each item in 3-year-old girls

Figures 8 and 9 showed the number of children for whom no concern was noted by professionals and the number of children whose parents were (or were not) worried about each item in 3-year-old boys and girls.

Among boys (Fig. 8), many items related to “Food experience and behavior” were indicated as not being of concern by professionals. Among these, a large proportion of parents were worried about "low experience of meal preparation," "not allowing a child to help with meals," and "low experience of growing crops." Further, among items related to "Interest and motivation in food", a large proportion of parents were worried about "inconsistent amount of food," “taking longer to eat” and “playing with food.” For girls (Fig. 9), many items related to “Food experience and behavior’’ were not considered to be concerns by professionals. In the category “Interest and motivation in food,” a large proportion of parents were concerned about “inconsistent amount of food,” “taking longer to eat,” and “playing with food.”

### The gap between professionals’ concerns and parents’ perceptions

Table 5 shows the differences between the group of 18-month-old boys for which the parents were concerned about items noted as concerns by professionals and the group for which the parents were not concerned for those items. The items more commonly noted by professionals as being of concern (≥ 10 professionals expressed the concern) were “nutritional balance of foods” (13 professionals), “inconsistent amount of food” (10 professionals), “taking longer to eat” (10 professionals), “playing with food” (17 professionals), and “picky eating” (17 professionals).

**Table 5:** Gap between the concerns diagnosed by health professionals and recognition of parents’ worries regarding children's diet　(Boys, 18 months, *n* = 94)

	Item	Health professionals		Health professionals						
		Number of children with concern	Parent’s level of concern	With concern n	Without concern n	Total n	Column proportion			Youden index
							*Sensitivity* FNR	FPR *Specificity*	total	
Health awareness and lifestyle	control of beverage^*^	5	worried	2	4	6	0.40	0.04	0.06	***0.36***
			not worried	3	85	88	***0*** ***.*** ***60***	*0.96*	0.94	
	control of time of snack	7	worried	5	3	8	*0.71*	0.03	0.09	0.68
			not worried	2	84	86	0.29	*0.97*	0.91	
	control of amount of snack	7	worried	5	7	12	0.71	0.08	0.13	0.59
			not worried	2	80	82	0.29	*0.92*	0.87	
	parent’s dietary life rhythm	1	worried	1	1	2	*1.00*	0.01	0.02	0.99
			not worried	0	92	92	0.00	*0.99*	0.98	
	control of bedtime/wake-up time	4	worried	4	9	13	*1.00*	0.10	0.14	0.90
			not worried	0	81	81	0.00	*0.90*	0.86	
	parent’s dietary lifestyle	1	worried	1	0	1	*1.00*	0.00	0.01	0.99
			not worried	0	93	93	0.00	*1.00*	0.99	
	not going to health checkups	1	worried	0	0	0	*0.00*	0.00	0.00	0.00
			not worried	1	93	94	1.00	*1.00*	1.00	
	how child is eating in school	1	worried	1	2	3	*1.00*	0.02	0.03	0.97
			not worried	0	91	91	0.00	*0.98*	0.97	
	low information on child's diet in the daytime	0	worried	0	1	1	*-*	0.xx	0.xx	-
			not worried	0	93	93	-	*0.xx*	0.xx	
	low awareness of diet	0	worried	0	4	4	*-*	0.xx	0.xx	-
			not worried	0	90	90	-	*0.xx*	0.xx	
Diet content and atmosphere	combination of ingredients	4	worried	4	10	14	*1.00*	0.11	0.15	0.87
			not worried	0	80	80	0.00	*0.89*	0.85	
	combination of foods	8	worried	8	16	24	*1.00*	0.19	0.26	0.80
			not worried	0	70	70	0.00	*0.81*	0.74	
	**nutritional balance of foods**†	**13**	worried	12	23	35	*0.92*	**0.28**	0.37	0.64
			not worried	1	58	59	0.08	*0.72*	0.63	
	**arrangements and colors of foods**†	3	worried	3	27	30	*1.00*	**0.30**	0.32	0.69
			not worried	0	64	64	0.00	*0.70*	0.68	
	knowledge of seasoning foods	4	worried	4	12	16	*1.00*	0.13	0.17	0.84
			not worried	0	78	78	0.00	*0.87*	0.83	
	not good at cooking	2	worried	2	12	14	*1.00*	0.13	0.15	0.84
			not worried	0	80	80	0.00	*0.87*	0.85	
	not eating with relish	0	worried	0	4	4	*-*	0.xx	0.xx	-
			not worried	0	90	90	-	*0.xx*	0.xx	
	eating only a few types of foods	0	worried	4	7	11	*0.00*	0.08	0.12	***-0.12***
			not worried	0	83	83	0.00	*0.92*	0.88	
Interest and motivation in food	small amount of food intake	1	worried	1	7	8	*1.00*	0.08	0.09	0.94
			not worried	0	86	86	0.00	*0.92*	0.91	
	not being hungry at meal time	3	worried	3	1	4	*1.00*	0.01	0.04	0.99
			not worried	0	90	90	0.00	*0.99*	0.96	
	**inconsistent amount of food**†	**10**	worried	15	19	34	*1.00*	**0.24**	0.36	***0.17***
			not worried	0	60	60	0.00	*0.76*	0.64	
	taking longer to eat	**10**	worried	9	12	21	*0.90*	0.14	0.22	0.76
			not worried	1	72	73	0.10	*0.86*	0.78	
	playing with food	**17**	worried	15	13	28	*0.88*	0.17	0.30	0.72
			not worried	2	64	66	0.12	*0.83*	0.70	
	**picky eating**†	**17**	worried	15	18	33	0.94	**0.23**	0.35	***-0.03***
			not worried	1	60	61	0.06	*0.77*	0.65	
	irregular meal times	3	worried	3	3	6	*1.00*	0.03	0.06	0.97
			not worried	0	88	88	0.00	*0.97*	0.94	
	spending much time watching media	8	worried	8	13	21	*1.00*	0.15	0.22	0.84
			not worried	0	73	73	0.00	*0.85*	0.78	
Food experience and behavior	low experience with meal preparation	8	worried	0	54	54	*-*	0.xx	0.xx	-
			not worried	0	40	40	-	*0.xx*	0.xx	
	**not allowing child to help with meals**†	1	worried	1	56	57	*1.00*	**0.60**	0.61	***0.37***
			not worried	0	37	37	0.00	*0.40*	0.39	
	low experience of growing crops	0	worried	0	49	49	*-*	0.xx	0.xx	-
			not worried	0	45	45	-	*0.xx*	0.xx	
	not being able to use a spoon and fork	3	worried	3	11	14	*1.00*	0.12	0.15	0.88
			not worried	0	80	80	0.00	*0.88*	0.85	
	The number of items with gaps between professionals and parents (total 30 items)						1*	**5**†		**5**‡

*FNR* False negative rate (1-sensitivity), *FPR* False positive rate (1-specificity)

Underlined value:

^*^FNR > 0.5 (More than half of parents were not worried about the item that were concerned by professionals)

^**†**^FPR > 0.2 (A high proportion of parents were worried about the item that were not concerned by professionals), or

^‡^Youden index < 0.5 (A high discordance were shown between professionals and parents)

Underlined item:

^*^FNR > 0.5 or ^†^FPR > 0.2

There was one item for which the professionals were concerned but more than half of parents were not (FNR > 0.5): “control of beverage” (0.60). In all, five items showed a difference between the groups in which the professionals were not concerned, but the parents were worried (FPR > 0.2), such as "nutritional balance of foods" (0.28), "arrangements and colors of foods" (0.30), "inconsistent amount of food" (0.24), "picky eating" (0.23), and "not allowing the child to help with meals" (0.60). For five items, parents’ perceptions differed more from those of the professionals (Youden index < 0.5), including “control of beverage” (0.36), “inconsistent amount of food” (0.17), “picky eating” (− 0.03), and "not allowing the child to help with meals" (0.37).

Table 6 presents the differences regarding whether the parents of 18-month-old girls were concerned with items noted as concerns by professionals.

**Table 6:** Gap between the concerns diagnosed by health professionals and by parents’ concerns regarding children’s diet (Girls, 18 months, *n* = 65)

	Item	Health professionals		Health professionals						
		Number of children with concern	Parent’s level of concern	With concern n	Without concern n	Total n	Column proportion			Youden index
							*Sensitivity* FNR	FPR *Specificity*	total	
Health awareness and lifestyle	control of beverage	7	worried	6	4	10	*0.86*	0.07	0.15	0.79
			not worried	1	54	55	0.14	*0.93*	0.85	
	control of time of snack	8	worried	7	8	15	*0.88*	0.14	0.23	0.73
			not worried	1	49	50	0.12	*0.86*	0.77	
	control of amount of snack	**10**	worried	8	10	18	*0.80*	0.18	0.28	0.62
			not worried	2	45	47	0.20	*0.82*	0.72	
	parent’s dietary life rhythm	1	worried	1	4	5	*1.00*	0.06	0.08	0.94
			not worried	0	60	60	0.00	*0.94*	0.92	
	control of bedtime/wake-up time	3	worried	3	6	9	*1.00*	0.10	0.14	0.90
			not worried	0	56	56	0.00	*0.90*	0.86	
	parent’s dietary lifestyle	1	worried	1	8	9	*1.00*	0.13	0.14	0.88
			not worried	0	56	56	0.00	*0.87*	0.86	
	not going to health checkups	2	worried	1	2	3	*0.50*	0.03	0.05	**0.47**
			not worried	1	61	62	0.50	*0.97*	0.95	
	how child is eating in school	1	worried	1	4	5	*1.00*	0.06	0.08	0.94
			not worried	0	60	60	0.00	*0.94*	0.92	
	low information on child’s diet in the daytime	0	worried	0	2	2	*-*	0.xx	0.xx	-
			not worried	0	63	63	-	*0.xx*	0.xx	
	low awareness of diet	2	worried	2	8	10	*1.00*	0.13-	0.15-	0.87
			not worried	0	55	55	-0.00	*0.87-*	0.85-	
Diet content and atmosphere	**combination of ingredients**†	5	worried	4	14	18	*0.80*	**0.23**	0.28	0.57
			not worried	1	46	47	0.20	*0.77*	0.72	
	**combination of foods†**	8	worried	7	13	20	*0.88*	**0.23**	0.31	0.65
			not worried	1	44	45	0.12	*0.77*	0.69	
	**nutritional balance of foods**†	**12**	worried	12	19	31	*1.00*	**0.36**	0.48	0.64
			not worried	0	34	34	0.00	*0.64*	0.52	
	**arrangements and colors of foods**†	3	worried	3	25	28	*1.00*	**0.40**	0.43	0.60
			not worried	0	37	37	0.00	*0.60*	0.57	
	knowledge of seasoning foods	1	worried	1	10	11	*1.00*	0.16	0.17	0.84
			not worried	0	54	54	0.00	*0.84*	0.83	
	not good at cooking	0	worried	0	13	13	*-*	0.xx	0.xx	-
			not worried	0	52	52	-	*0.xx*	0.xx	
	not eating will relish	0	worried	0	4	4	*-*	0.xx	0.xx	-
			not worried	0	61	61	-	*0.xx*	0.xx	
	**eating only a few types of foods**†	2	worried	2	12	14	*1.00*	0.19	**0.22**	0.81
			not worried	0	51	51	0.00	*0.81*	0.78	
Interest and motivation in food	small amount of food intake	3	worried	3	4	7	*1.00*	0.06	0.11	0.94
			not worried	0	58	58	0.00	*0.94*	0.89	
	not being hungry at meal time	4	worried	4	1	5	*1.00*	0.02	0.08	0.98
			not worried	0	60	60	0.00	*0.98*	0.92	
	**inconsistent amount of food**†	**12**	worried	12	22	34	*1.00*	**0.42**	0.52	0.58
			not worried	0	31	31	0.00	*0.58*	0.48	
	**taking longer to eat**†	4	worried	4	16	20	*1.00*	**0.26**	0.31	0.74
			not worried	0	45	45	0.00	*0.74*	0.69	
	playing with food	**11**	worried	11	20	31	*1.00*	**0.37**	0.48	0.63
			not worried	0	34	34	0.00	*0.63*	0.52	
	**picky eating**†	**13**	worried	10	15	25	*0.77*	**0.29**	0.38	**0.48**
			not worried	3	37	40	0.23	*0.71*	0.62	
	Irregular meal times	1	worried	1	6	7	*1.00*	0.09	0.11	0.91
			not worried	0	58	58	0.00	*0.91*	0.89	
	spending much time watching media	**11**	worried	11	10	21	*1.00*	0.19	0.32	0.81
			not worried	0	44	44	0.00	*0.81*	0.68	
Food experience and behavior	low experience of meal preparation	0	worried	0	44	44	*-*	0.xx	0.xx	-
			not worried	0	21	21	-	*0.xx*	0.xx	
	**not allowing child to help with meals**^b^	1	worried	1	45	46	*1.00*	**0.70**	0.71	**0.30**
			not worried	0	19	19	0.00-	*0.30*	0.29	
	low experience of growing crops	0	worried	0	36	36	*-*	0.xx	0.xx	-
			not worried	0	29	29	-	*0.xx*	0.xx	
	not being able to use spoon and fork	1	worried	1	8	9	*1.00*	0.13	0.14	0.88
			not worried	0	56	56	0.00	*0.87*	0.86	
	The number of items with gaps between professionals and parents (total 30 items)						0*	**8**†		**3**‡

*FNR* False negative rate (1-sensitivity), *FPR* False positive rate (1-specificity)

Underlined value:

^*^FNR > 0.5 (More than half of parents were not worried about the item that were concerned by professionals)

^†^FPR > 0.2 (A high proportion of parents were worried about the item that were not concerned by professionals), or

^‡^Youden index < 0.5 (A high discordance were shown between professionals and parents)

Underlined item:

^†^FPR > 0.2

The items of concern to professionals (≥ 10 professionals expressed the concern) were “control of amount of snack” (10 professionals), “nutritional balance of foods” (12 professionals), “inconsistent amount of food” (12 professionals), “playing with food” (11 professionals), “picky eating” (13 professionals), and “spending too much time watching media” (11 professionals).

There were no items for which more than half of parents were not concerned and were objects of concern by professionals (FNR > 0.5).

There were eight items for which professionals were not concerned but parents were worried (FPR > 0.2), including "combination of foods" (0.23), "nutritional balance of foods" (0.36), "arrangements and colors of foods" (0.40), "inconsistent amount of food" (0.42), "picky eating” (0.29), and "not allowing the child to help with meals" (0.70).

The items for which parents’ perceptions differed more from those of the professionals (Youden index < 0.5) were 3 items, including “picky eating” (0.48), and “not allowing child to help with meals” (0.30).

Table 7 presents items for which the parents were concerned and were noted as concerns by professionals for 3-year-old boys.

**Table 7:** Gap between the concerns diagnosed by health professionals and parents’ concerns regarding children’s diet　(Boys, 3 years, *n* = 70)

	Item	Health professionals		Health professionals						
		Number of children with concern	Parent’s level of concern	With concern n	Without concern n	Total n	Column proportion			Youden index
							*Sensitivity* FNR	FPR *Specificity*	total	
Health awareness and lifestyle	control of beverage	6	worried	5	8	13	*0.83*	0.13	0.19	0.71
			not worried	1	56	57	0.17	*0.88*	0.81	
	control of time of snack	5	worried	4	8	12	*0.80*	0.12	0.17	0.68
			not worried	1	57	58	0.20	*0.88*	0.83	
	control of amount of snack	6	worried	5	12	17	*0.83*	0.19	0.24	0.65
			not worried	1	52	53	0.17	*0.81*	0.76	
	parent’s dietary life rhythm	1	worried	1	6	7	*1.00*	0.09	0.10	0.91
			not worried	0	63	63	0.00	*0.91*	0.90	
	control of bedtime/wake-up time	**10**	worried	9	12	21	*0.90*	0.20	0.30	0.70
			not worried	1	48	49	0.10	*0.80*	0.70	
	parent’s dietary lifestyle	0	worried	0	6	6	*-*	0.xx	0.xx	-
			not worried	0	64	64	-	*0.xx*	0.xx	
	not going to health checkups	2	worried	0	5	5	*0.00*	0.07	0.07	**-0.07**
			not worried	2	63	65	1.00	*0.93*	0.93	
	how child is eating in school	1	worried	1	9	10	*1.00*	0.13	0.14	0.87
			not worried	0	60	60	0.00	*0.87*	0.86	
	**l**ow information on child’s diet in daytime	0	worried	0	8	8	*-*	0.xx	0.xx	-
			not worried	0	62	62	-	*0.xx*	0.xx	
	low awareness of diet	0	worried	0	12	12	*-*	0.xx	0.xx	-
			not worried	0	58	58	-	*0.xx*	0.xx	
Diet content and atmosphere	**combination of ingredients**†	3	worried	3	16	18	*1.00*	**0.24**	0.26	0.76
			not worried	0	51	51	0.00	*0.76*	0.73	
	**combination of foods**†	8	worried	7	20	27	*0.88*	**0.32**	0.39	0.55
			not worried	1	42	43	0.13	*0.68*	0.61	
	**nutritional balance of foods**†	**10**	worried	10	25	35	*1.00*	**0.42**	0.50	0.58
			not worried	0	35	35	0.00	*0.58*	0.50	
	arrangements and colors of foods*†	3	worried	3	24	27	*0.43*	**0.36**	0.39	0.64
			not worried	4	43	43	**0.57**	*0.64*	0.61	
	knowledge of seasoning foods	1	worried	1	11	12	*1.00*	0.16	0.17	0.84
			not worried	0	58	58	0.00	*0.84*	0.83	
	**not good at cooking**†	1	worried	1	28	29	*1.00*	**0.41**	0.41	0.59
			not worried	0	41	41	0.00	*0.59*	0.59	
	not eating with relish	1	worried	0	5	5	*0.00*	0.07	0.07	**-0.07**
			not worried	1	64	65	1.00	*0.93*	0.93	
	**eating only few types of foods**†	5	worried	5	15	20	*1.00*	**0.23**	0.29	0.77
			not worried	0	50	50	0.00	*0.77*	0.71	
Interest and motivation in food	**small amount of food intake†**	5	worried	5	16	21	*1.00*	**0.25**	0.30	0.75
			not worried	0	49	49	0.00	*0.75*	0.70	
	not being hungry at meal time	3	worried	3	8	11	*1.00*	0.12	0.16	0.88
			not worried	0	59	59	0.00	*0.88*	0.84	
	**inconsistent amount of food**†	**19**	worried	18	31	49	*0.95*	**0.61**	0.70	**0.34**
			not worried	1	20	21	0.05	*0.39*	0.30	
	**taking longer to eat**†	**18**	worried	18	28	46	*1.00*	**0.54**	0.66	0.46
			not worried	0	24	24	0.00	*0.46*	0.34	
	**playing with food**†	**18**	worried	17	27	44	*0.94*	**0.52**	0.63	**0.43**
			not worried	1	25	26	0.06	*0.48*	0.37	
	**picky eating**†	**16**	worried	16	18	34	*1.00*	**0.33**	0.49	0.67
			not worried	0	36	36	0.00	*0.67*	0.51	
	irregular meal times	0	worried	0	9	9	*-*	0.xx	0.xx	-
			not worried	0	61	61	-	*0.xx*	0.xx	
	**spending too much time watching media**†	6	worried	6	31	37	*1.00*	**0.48**	0.53	0.52
			not worried	0	33	33	0.00	*0.52*	0.47	
Food experience and behavior	**low experience of meal preparation**†	1	worried	1	23	24	*1.00*	**0.33**	0.34	0.67
			not worried	0	46	46	0.00	*0.67*	0.66	
	**not allowing child to help with meals**†	2	worried	2	33	35	*1.00*	**0.49**	0.50	0.51
			not worried	0	35	35	*0.00*	*0.51*	0.50	
	low experience of growing crops	0	worried	0	25	25	*-*	0.xx	0.xx	-
			not worried	0	45	45	-	*0.xx*	0.xx	
	not being able to use spoon and fork	0	worried	0	0	0	*-*	0.xx	0.xx	-
			not worried	0	70	70	-	*0.xx*	0.xx	
	The number of items with gaps between professionals and parents (total 30 items)						1*	**14**†		**4**‡

*FNR* False negative rate (1-sensitivity), *FPR* False positive rate (1-specificity)

Underlined value:

^*^FNR > 0.5 (More than half of parents were not worried about the item that were concerned by professionals)

^†^FPR > 0.2 (A high proportion of parents were worried about the item that were not concerned by professionals), or

^‡^Youden index < 0.5 (A high discordance were shown between professionals and parents)

Underlined item:

^*^FNR > 0.5 or ^†^FPR > 0.2

Among the items noted by professionals as being of concern, the more frequently cited items (≥ 10 professionals expressed the concern) were “control of bedtime/wake-up time” (10 professionals), “nutritional balance of foods” (10 professionals), “inconsistent amount of food” (19 professionals), “taking longer to eat” (18 professionals), “playing with food” (18 professionals) and “picky eating” (16 professionals)."

There was one item for which the professionals were concerned but more than half of parents were not (FNR > 0.5): “arrangements and colors of foods” (0.57).

For 14 items, the professionals were not concerned but the parents were (FPR > 0.2), such as "combination of foods" (0.24), "nutritional balance of foods" (0.42), "arrangements and colors of foods" (0.36), "eating only a few types of foods" (0.23), "inconsistent amount of food" (0.61), "taking longer to eat” (0.54), “playing with food (0.52), “picky eating” (0.33), “spending too much time watching media” (0.48), and “not allowing the child to help with meals” (0.49).

Four items for which parents’ perceptions differed more from those of the professionals (Youden index < 0.5) included "inconsistent amount of food" (0.34)" and “playing with food” (0.43).

Tables 8 shows the differences between the two groups where the parents were worried regarding items noted as concerns by professionals for 3-year-old girls.

**Table 8:** Gap between the concerns diagnosed by health professionals and parents’ concerns regarding children's diet　(Girls, 3 years, n = 76)

	Item	Health professionals		Health professionals						
		Number of children with concern	Parent’s level of concern	With concern n	Without concern n	Total n	Column proportion			Youden index
							*Sensitivity* FNR	FPR *Specificity*	total	
Health awareness and lifestyle	control of beverage	8	worried	8	8	16	*1.00*	0.12	0.21	0.88
			not worried	0	60	60	0.00	*0.88*	0.79	
	control of time of snack	7	worried	7	6	13	*1.00*	0.09	0.17	0.91
			not worried	0	63	63	0.00	*0.91*	0.83	
	control of amount of snack	**13**	worried	13	11	24	*1.00*	0.17	0.32	0.83
			not worried	0	52	52	0.00	*0.83*	0.68	
	parent's dietary life rhythm	2	worried	1	7	8	*0.50*	0.09	0.11	**0.41**
			not worried	1	67	68	0.50	*0.91*	0.89	
	control of bedtime/wake-up time	**12**	worried	12	7	19	*1.00*	0.11	0.25	0.89
			not worried	0	57	57	0.00	*0.89*	0.75	
	parent's dietary lifestyle	1	worried	1	3	4	*1.00*	0.04	0.05	0.96
			not worried	0	72	72	0.00	*0.96*	0.95	
	not going to health checkups	3	worried	2	0	2	*0.67*	0.00	0.03	0.67
			not worried	1	73	74	0.33	*1.00*	0.97	
	how child is eating in school	1	worried	1	4	5	*1.00*	0.05	0.07	0.95
			not worried	0	71	71	0.00	*0.95*	0.93	
	low information of child’s diet in daytime	2	worried	2	3	5	*1.00*	0.04	0.07	0.96
			not worried	0	71	71	0.00	*0.96*	0.93	
	low awareness of diet	0	worried	0	7	10	*-*	0.xx	0.xx	-
			not worried	0	69	75	-	*0.xx*	0.xx	
Diet content and atmosphere	combination of ingredients	4	worried	4	10	14	*1.00*	0.14	0.18	0.86
			not worried	0	62	62	0.00	*0.86*	0.82	
	combination of foods**†**	5	worried	5	15	20	*1.00*	**0.21**	0.26	0.79
			not worried	0	56	56	0.00	*0.79*	0.74	
	**nutritional balance of foods**†	**13**	worried	13	24	37	*1.00*	**0.38**	0.49	0.62
			not worried	0	39	39	0.00	*0.62*	0.51	
	**arrangements and colors of foods**†	2	worried	2	16	18	*1.00*	**0.22**	0.24	0.78
			not worried	0	58	58	0.00	*0.78*	0.76	
	knowledge of seasoning foods	1	worried	1	5	6	*1.00*	0.07	0.08	0.93
			not worried	0	70	70	0.00	*0.93*	0.92	
	**not good at cooking**†	2	worried	2	18	20	*1.00*	**0.24**	0.26	0.76
			not worried	0	56	56	0.00	*0.76*	0.74	
	not eating with relish	0	worried	0	1	1	*-*	0.xx	0.xx	-
			not worried	0	75	75	-	*0.xx*	0.xx	
	eating only few types of foods	4	worried	4	11	15	*1.00*	0.15	0.20	0.85
			not worried	0	61	61	0.00	*0.85*	0.80	
Interest and motivation in food	**small amount of food intake**†	6	worried	6	16	22	*1.00*	**0.23**	0.29	0.77
			not worried	0	54	54	0.00	*0.77*	0.71	
	not being hungry at meal time	5	worried	5	6	11	*1.00*	0.08	0.14	0.92
			not worried	0	65	65	0.00	*0.92*	0.86	
	**inconsistent amount of food**†	**24**	worried	24	28	52	*1.00*	**0.54**	0.68	**0.46**
			not worried	0	24	24	0.00	*0.46*	0.32	
	**taking longer to eat**†	**22**	worried	22	28	50	*1.00*	**0.52**	0.66	**0.48**
			not worried	0	26	26	0.00	*0.48*	0.34	
	**playing with food**†	**13**	worried	13	30	43	*1.00*	**0.48**	0.57	0.52
			not worried	0	33	33	0.00	*0.52*	0.43	
	**picky eating**†	**13**	worried	12	15	27	*0.92*	**0.24**	0.36	0.68
			not worried	1	48	49	0.08	*0.76*	0.64	
	irregular meal times	1	worried	1	6	7	*1.00*	0.08	0.09	0.92
			not worried	0	69	69	0.00	*0.92*	0.91	
	**spending much watching media**†	**13**	worried	13	26	39	*0.93*	**0.42**	0.51	0.51
			not worried	1	36	37	0.07	*0.58*	0.49	
Food experience and behavior	low experience of meal preparation	0	worried	0	13	13	*-*	0.17	0.17	**-0.17**
			not worried	0	62	63	-	*0.83*	0.83	
	**not allowing child to help with meals**†	1	worried	1	19	20	*1.00*	**0.25**	0.26	0.75
			not worried	0	56	56	0.00	*0.75*	0.74	
	**l**ow experience of growing crops	0	worried	0	23	23	-	0.xx	0.xx	-
			not worried	0	53	53	-	*0.xx*	0.xx	
	not being able to use spoon and fork	1	worried	1	0	1	*1.00*	0.00	0.01	1.00
			not worried	0	75	75	0.00	*1.00*	0.99	
	The number of items with gaps between professionals and parents (total 30 items)						0*	**11**†		**4**‡

*FNR* False negative rate (1-sensitivity), *FPR* False positive rate (1-specificity)

Underlined value:

^*^FNR > 0.5 (More than half of parents were not worried the item that were concerned by professionals)

^†^FPR > 0.2 (A high proportion of parents were worried about the item that were not concerned by professionals), or

^‡^Youden index < 0.5 (A high discordance were shown between professionals and parents)

Underlined item:

^†^FPR > 0.2

Among the items indicated by professionals to be of concern, the more frequently cited items (≥ 10 professionals expressed a concern) included "control of the amount of snack (including sweets)" (13 professionals), “control of bedtime/wake-up time” (12 professionals), “nutritional balance of foods” (13 professionals), “inconsistent amount of food” (24 professionals), “taking longer to eat” (22 professionals), “playing with food” (13 professionals), “picky eating” (13 professionals), and “spending too much time watching media” (13 professionals).

There was no item for which more than half of parents were not concerned and professionals were concerned (FNR > 0.5).

There were 11 items where a difference between the groups whereby the professionals were not concerned but the parents were (FPR > 0.2), including “nutritional balance of foods” (0.38), “inconsistent amount of food” (0.54), “taking longer to eat” (0.52), “playing with food” (0.48), “picky eating” (0.24), and “spending much time watching media” (0.42).

For four items, parents’ perceptions differed from those of professionals (Youden index < 0.5) were included “inconsistent amount of food” (0.46)” and “taking longer to eat” (0.48).

## Discussion

In this study, it was identified the discrepancies between the opinions of professionals and the perceptions of parents regarding dietary concerns for preschool children. Previous studies have reported that picky eating and eating unbalanced diets including snacks and beverages are important issues for preschool children and tend to have gaps in perceptions between professionals and parents [7, 19, 20, 34, 35]. However, few reports have identified differences between professionals' and parents' concerns regarding the age and gender of children.

Our study was conducted among boys and girls aged 18 months and 3 years old. Among the notable findings of the study, for both 18-month- and 3-year-old children, many parents were concerned about issues that professionals did not consider concerning (FPR > 0.2). Moreover, the number of items that parents worried about (FPR > 0.2) for 3-year-olds was higher than for 18-month-olds.

On the other hand, although, “control of beverage” for 18-month-old boys was not an item of concern for some parents, professionals indicated that this could be a concern for them. In other words, it was noted that parents’ concerns differed by gender and age of their children.

In the results of this study, the items for which ≥ 10 professionals indicated concerns and with a higher proportion of discordance between the professionals and parents for both boys and girls were "picky eating" in 18-month-olds and “inconsistent amount of food” in 3-year-olds.

The relationship between “picky eating” and “poor dietary habits” in children has been reported before [36, 37].

In this study, it was identified that some parents do not correctly recognize these matters in their children. Dietitians, public health nurses, and other professionals should understand the gap between parents’ perceptions and their own.

In addition, it was confirmed that in the group of boys with professional concerns, mean birth height and birth weight were lower than in those without concerns. Professionals need to provide long-term counseling and support these parents and children.

Previous studies that have noted the contrast between parents’ and professionals’ concerns have indicated the difficulty of getting parents to understand the concepts and terminology related to child nutrition as used by professionals [12, 32, 34]; for example, understanding growth through height and weight measurements [38] and the importance of continuous life care from beginning before childbirth to childhood. [39] It should be noted that although many parents obtain childcare support information from the Internet, they may not be receiving it from childcare professionals [40]. Parents may not recognize how many sweet beverages their children are drinking because they have insufficient knowledge of nutritional balance.

Another reason for the discrepancy may be parents tend to only want information on how to deal with the situation of their children. Efforts should be made to ensure that information from healthcare professionals can lead to parental knowledge and practical skills, including cooking skills, and both parents and professionals can work together to improve the quality of meals for children’s healthy development [12, 41, 42]. It is also suggested that parents and professionals may have different interpretations of diets and meal preparation [8, 43], and the understanding of diets and meal preparation may be related to the parental childcare environment and parent–child communication [44].

For the 3-year-old children, there were more common items that professionals considered to be as concerning in the first-born children than in the second- and subsequent-born children. Appropriate advice or nutrition education from professionals may be necessary to let parents understand the dietary issues from a broad perspective, including the child’s birth order and relationship with their brothers and sisters and with their parents. However, there have been few reports on the degree of understanding of parents involved in dietary care with respect to nutritional guidance, and future research is necessary.

For children for whom concerns were noted by professionals, many have working mothers [45] and are therefore sent to nursery schools or their grandparents' homes during the day. A previous study reported on children’s dietary issues in Japan found that children in households where mothers work tend to skip breakfast and have poor control of snacks; this suggested that children’s poorly balanced diet is related to the low awareness of the parent’s own diet and eating habits [46]. However, in this study, few parents answered that they had problems with their eating habits. Taking into account the working situation of the parents, it is necessary to consider how to proceed with childcare and nutrition consultation for working parents, and it may be also necessary to find the gaps between the perceptions of parents and professionals on the issues.

For nutritional improvement with the life course perspective of the child, if there are incorrect perceptions in parents must be corrected to influence the quality of children's feeding [47].

To that end, instead of giving guidance assuming a uniform ideal situation that focuses only on the parents and family living together, a broader look at everyday reality should be taken and the child’s siblings, friends, and peers who spend the day together with them. It should be navigating the achievable goals of individual caregivers by professionals [12, 13, 48].

Several limitations of this study should be addressed.

First, the three municipalities showed different cooperation rates. The reason why it was difficult to obtain cooperation in some municipalities was the number of children coming for a health checkup was in some cases very large, and the professionals were very busy with their duties, making it difficult to respond to our survey.

Future studies should take this time-based aspect into account.

Second, cooperation from populous urban municipalities could not be obtained. In larger settlements, health checkups are outsourced to the private sector, and temporary workers are often involved in health checkups, making it difficult to coordinate standardized survey methods. In the future, it will be necessary to examine the survey method at the time of health checkups for large city-type municipalities.

Third, although the cooperation rate was high, some items had few responses from parents, in particular regarding income, height at birth, and weight at birth. Some parents often entrusted the maternal and child health handbook to the municipality staff before health checkups, so they did not have it at hand and could not obtain data recorded in it, including the child’s birth height and weight. The income was difficult to answer. It will be necessary to examine the study methodology to improve these issues in the future.

Another limitation regarded how the gaps between the professionals’ and the parents’ concerns revealed in these analyses affects the children’s health and nutritional status. Further research is needed on this issue.

However, it was found that the gaps between the professionals’ and parents’ concerns differed by age and gender of children. It is necessary to investigate how to proceed with childcare and nutrition counseling by professionals to allow parents to correctly recognize potential issues in their children’s eating in early childhood.

## Conclusion

This study investigated gaps between the concerns of professionals and the concerns of parents regarding the health and dietary habits of their preschool children. A gap was seen between the concerns noted by professionals and those perceived by parents. For the children for whom professionals had concerns, this was more common in the first-born children than in the second- and subsequent-born children. For several items, the parents expressed concern regarding items that the professionals did not consider concerning. The items for which ≥ 10 professionals indicated concerns and with higher discordance between the professionals and parents for both boys and girls were "picky eating" for 18-month-olds and "inconsistent amount of food" for 3-year-olds. For parents to develop a correct understanding of their children's food habits, it might be necessary to consider how to provide professional nutrition counseling for them.

### Supplementary Information

**Additional file 1.** Questionnaire.

## Data Availability

The datasets created and analyzed during the present study are available from the corresponding author upon reasonable request.

## Abbreviations

BMI: Body mass index
SD: Standard deviation
MHLW: Ministry of Health, Labour and Welfare
