# Supplementary material for: Gap between the concerns of healthcare professionals and parents’ perceptions regarding dietary habits for 18-month- and 3-year-old children in Japan
Source: BMC Public Health. 2023 Sep 30;23:1891. doi: 10.1186/s12889-023-16743-z (PMC10544123; doi:10.1186/s12889-023-16743-z)
Supplement: Supplementary file 1 — Additional file 1. Questionnaire. [file 12889_2023_16743_MOESM1_ESM.pdf]

## Questionnaire

| ID nunber 【　　】 | Please circle the gender of your child.　（ Boy　　Girl ） | Answer | | This section is to be filled out by healthcare professionals<br>Please fill in the profession and check any concerns. |
|---|---|---|---|---|
| | **Do you have any of the following concern about your child's diet, eating habits, or health? (A concern is a concern for the present situation, not for the future.)** | **Please circle yes or no.** | | （ ） P: Public health nurse<br>（ ） R: Registered dietitian<br>（ ） O: Other professionals |
| Health awareness and lifestyle | The parent feels a lack of control over types and amounts of child's beverages (including sweet drinks). | Yes | No | （ ） |
| | The parent feels a lack of control of their child's snack intake, frequency, and time. | Yes | No | （ ） |
| | The parent is unable to manage the types and amounts of child's snacks (including sweets). | Yes | No | （ ） |
| | The parent has problems with his or her dietary life rhythms (frequency and time). | Yes | No | （ ） |
| | The parent has a lack of control of bedtime/wake-up time. | Yes | No | （ ） |
| | The parentt has problems with his or her dietary lifestyle. | Yes | No | （ ） |
| | Child is not receiving ongoing health checks or rehabilitation for child development. | Yes | No | （ ） |
| | The parent did not understand what meals their child is eating at nursery school. | Yes | No | （ ） |
| | There is low information provided by the nursery school about the child's diet in the daytime. | Yes | No | （ ） |
| | Parent has problems with low awareness of diet (including meal preparation). | Yes | No | （ ） |
| Diet content and atmosphere | The type and combination of food and ingredients are unbalanced. | Yes | No | （ ） |
| | The type and combination of dishes (staple food, main dish, side dish) are not good. | Yes | No | （ ） |
| | The nutritional balance of foods is not good. | Yes | No | （ ） |
| | The arrangements and colors of food are not good. | Yes | No | （ ） |
| | The parent lack knowledge of seasoning foods. | Yes | No | （ ） |
| | The parent was not good at cooking meals. | Yes | No | （ ） |
| | My child does not eat with relish. | Yes | No | （ ） |
| | My child eats only few type of foods. | Yes | No | （ ） |
| Interest and motivation in food | The amount of food my child eats is always small. | Yes | No | （ ） |
| | My child is not hungry at mealtimes. | Yes | No | （ ） |
| | My child eating habits are not constant. | Yes | No | （ ） |
| | My child eats sluggishly (it takes a longer time to eat). | Yes | No | （ ） |
| | My child plays with his or her food (lazy eating). | Yes | No | （ ） |
| | My child has picky eating (unbalanced diet). | Yes | No | （ ） |
| | My child has irregular meal times. | Yes | No | （ ） |
| | My child has spent much watching media. | Yes | No | （ ） |
| Food experience and behavior | My child has low experience of meal preparation. | Yes | No | （ ） |
| | My child is not allowing child's to experience preparing meals(helping). | Yes | No | （ ） |
| | My child is not allowing them to experience the cultivating and harvesting of ingredients. | Yes | No | （ ） |
| | My child is not being able to use a spoon and fork. | Yes | No | （ ） |
